# Integrating gender medicine into modern healthcare: Progress and barriers

**DOI:** 10.1111/eci.70089

**Published:** 2025-06-12

**Authors:** Rubén Fuentes Artiles, Caroline E. Gebhard, Catherine Gebhard

**Affiliations:** ^1^ Department of Cardiology Bern University Hospital Inselspital, University of Bern Bern Switzerland; ^2^ Intensive Care Unit, Department of Acute Medicine University Hospital Basel Basel Switzerland; ^3^ Department of Nuclear Medicine University Hospital Zurich Zurich Switzerland; ^4^ Center for Molecular Cardiology University of Zurich Schlieren Switzerland

**Keywords:** education, gender medicine, medical practice, women's health

## Abstract

**Background:**

Sex and gender are fundamental determinants of health, influencing disease risk, diagnosis, treatment, and outcomes across medical disciplines. While sex refers to biological characteristics, gender encompasses sociocultural dimensions, including behaviours and identities.

**Results:**

The field of gender medicine has evolved significantly from its roots in the women's health movement of the 1960s and 1970s, which initially sought to address reproductive rights and the systematic exclusion of women from clinical research. Over time, the focus has expanded to recognize sex‐ and gender‐based differences in all populations, including men and gender‐diverse individuals. Despite progress, persistent challenges remain. Many clinical guidelines inadequately incorporate sex and gender considerations, and women continue to be underrepresented in clinical trials, resulting in suboptimal efficacy and a higher incidence of adverse effects in women. Recent initiatives, including government‐funded research programs, specialized gender medicine professorships and regulatory measures promoting equitable clinical trial participation, represent positive steps forward. However, a systematic, interdisciplinary approach is required to fully integrate gender‐sensitive medicine into research, education and clinical practice. This narrative review explores the historical development of gender medicine, current advancements and remaining challenges. We highlight the need for improved research methodologies, policy changes and targeted interventions to ensure equitable healthcare. A structured action plan emphasizing regulatory support, education, industry involvement and public awareness is essential to accelerate the field's integration.

**Conclusion:**

Recognising and addressing sex‐ and gender‐sensitive health differences will lead to more personalised and effective medical care, ultimately improving health outcomes for all individuals.

## Graphical abstract

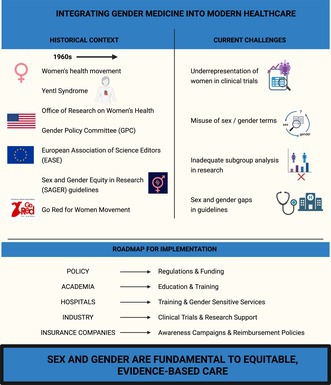

## 1 INTRODUCTION

Sex and gender are increasingly recognized as crucial determinants of health across all medical disciplines.^1^ While ‘sex’ pertains to biological characteristics rooted in genetics, hormones and reproductive anatomy, ‘gender’ refers to sociocultural dimensions, including attitudes, behaviours and identities.^2^ The field of gender medicine has evolved significantly from its origins during the women's health movement in the 1960s and 1970s, when advocacy for reproductive rights and equal inclusion in clinical research emerged prominently. Early exclusion of women from clinical trials, due to misconceptions about hormonal fluctuations and potential pregnancy risks, highlighted severe gaps and inequalities in healthcare.^3^, ^4^, ^5^ The pioneering efforts of biomedical researchers, such as Marianne Legato and Bernadine Healy, have propelled gender medicine forward, advocating for recognition of sex and gender differences beyond reproductive health alone.^6^, ^7^

Today, the field faces new challenges and opportunities. Despite advancements, clinical guidelines often still inadequately incorporate sex‐ and gender‐sensitive considerations, leading to continued disparities in diagnosis, treatment, and outcomes across diverse populations. Recent initiatives, including the establishment of dedicated women's health centres, specialised professorships in gender medicine and government‐funded research programmes, indicate positive momentum. However, broader interdisciplinary cooperation, systematic integration of gender‐sensitive approaches in research, education, and clinical routine, and the adoption of robust statistical methodologies to address sex and gender differences remain essential. This narrative review aims to critically analyse historical developments, highlight current challenges and outline future strategies necessary for fully integrating gender medicine into everyday clinical practice and research.

## 2 HISTORICAL ASPECTS: THE WOMEN'S HEALTH MOVEMENT

Gender medicine has evolved substantially since its beginnings in the women's health movement of the 1960s and 1970s. Initially, significant attention was drawn to the systematic exclusion of women from clinical trials.^3^ This exclusion was rooted in concerns that hormonal fluctuations could complicate study outcomes, assumptions that male results were universally applicable, and fears related to undetected pregnancies.^4^, ^5^, ^8^ These concerns were particularly intensified by the thalidomide disaster, which underscored the severe risks of not adequately testing medications in women.^9^ Biomedical researcher Marianne Legato was instrumental in pioneering studies that investigated sex differences in cardiovascular health, challenging prevailing assumptions that men's and women's health differed only in reproductive function.^10^ Her advocacy helped broaden public and scientific understanding that gender medicine addressed health disparities across all genders.^6^ Bernadine Healy further amplified awareness through her concept of ‘Yentl Syndrome,’ a term she coined to describe the phenomenon whereby women are less likely to receive appropriate diagnostic and therapeutic interventions for heart disease unless they present with the same symptoms as men. The term references the character Yentl from the short story by Isaac Bashevis Singer—later adapted into a film by Barbra Streisand—who disguises herself as a man to access education. This metaphor captures how women must conform to male norms to receive equitable medical care.^7^ Concurrently, institutional responses such as those by the US National Institutes of Health (NIH), including the establishment of the Office of Research on Women's Health, played a critical role in integrating women more systematically into clinical research and medical education.^11^

Over subsequent decades, the focus of gender medicine expanded beyond biological sex differences, increasingly recognizing the significance of gender as a sociocultural construct.^2^ This shift has led to greater attention to men's health issues, notably in the context of mental health, and the adoption of intersectionality, emphasizing the complex interactions among sex, gender, ethnicity, age, and socioeconomic status.^12^, ^13^ Major milestones in formalizing these dimensions in medical research were the establishment of the Gender Policy Committee (GPC) by the European Association of Science Editors (EASE) in 2012 and the publication of the Sex and Gender Equity in Research (SAGER) guidelines in 2016 with later adaptions in 2022, providing structured methodologies for consistently addressing sex and gender considerations in medical studies.^14^, ^15^ Finally, the “Go Red for Women” initiative, launched nearly two decades ago, significantly raised public awareness about cardiovascular disease (CVD) as the leading cause of death among women, promoting education, preventive measures and advocacy efforts specifically tailored to women's heart health. The campaign contributed substantially to increased recognition of sex and gender differences in symptoms, diagnosis and treatment of heart disease.^16^

## 3 CURRENT DEVELOPMENTS: ADVANCEMENTS AND ONGOING CHALLENGES

**FIGURE 1:**
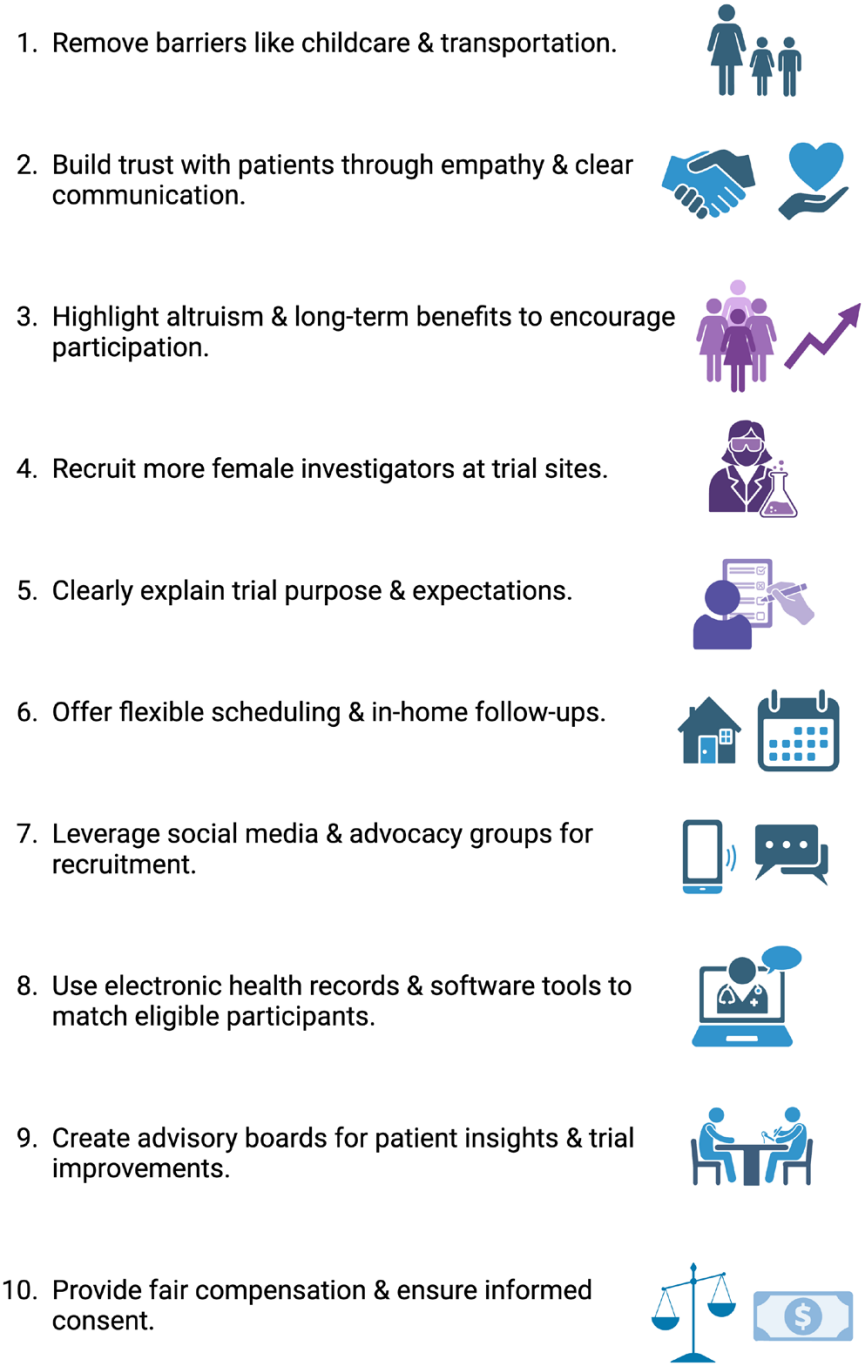
Strategies to improve the representation of women in clinical trials.

Nearly a decade after the introduction of the SAGER Guidelines, significant advancements have been made in gender medicine; however, considerable gaps persist. Many clinical guidelines still inadequately or superficially address sex‐ and gender‐sensitive considerations.^17^ A critical factor contributing to this issue is the continued underrepresentation of women in clinical trials. This is particularly problematic given growing evidence demonstrating substantial differences between males and females in drug absorption, distribution, metabolism and elimination, primarily due to physiological and hormonal variations.^18^, ^19^, ^20^ These differences significantly contribute to the higher incidence of adverse drug reactions (ADRs) observed in women.^21^, ^22^ The implications of insufficient female representation in clinical trials span across medical disciplines. Notable recent examples include Alzheimer's disease (AD) clinical trials, where sex‐specific treatment effects were not adequately analysed, despite women constituting the majority of AD patients.^23^ Similarly, the cardiovascular‐focused DANCANVAS trial exclusively recruited male participants, despite cardiovascular disease (CVD) being the leading cause of death among women.^24^ These examples highlight the pressing need for better representation and analysis of sex differences in clinical research to ensure equitable and effective healthcare for all genders. A positive example of efforts to enhance female participation in clinical trials is the ROMA:Women trial, the first dedicated cardiac surgery study designed to evaluate the outcomes of multiple arterial grafts for coronary artery bypass grafting specifically in women.^25^ This trial employs innovative strategies, including targeted recruitment, active involvement of patient representatives, and optimization of existing trial infrastructure to enhance enrollment and relevance. If successful, it could set a precedent for future gender‐sensitive cardiovascular trials. Additional strategies to improve female representation in clinical trials are outlined in Figure 1. Progress in drug development regarding gender includes growing recognition by pharmaceutical companies of the economic potential in ‘femtech’—the development of products specifically tailored to women's health needs. This reflects a gradual but significant shift toward greater gender inclusivity in medicine development. The World Economic Forum (WEF) has recently highlighted the rapid expansion of the ‘femtech’ sector, emphasising women's health as an area of substantial unmet need and considerable economic opportunity.^26^ The WEF advocates for increased investment in women's healthcare, positioning it as both a moral imperative and a lucrative market, with the potential to significantly boost global economic productivity and well‐being.^26^, ^27^ In parallel, countries such as Switzerland, the US, Canada and several European nations are actively working to integrate gender considerations into drug research and regulation through targeted measures. In 1993, the FDA mandated the inclusion of women in all phases of clinical drug development, reversing earlier policies that excluded women of childbearing age due to safety concerns. By 2025, the FDA will require comprehensive diversity plans for all Phase III clinical trials, ensuring well‐defined rationales for participant recruitment by sex and explicit gender‐based data analysis. Similarly, Canada and Europe have systematically incorporated gender considerations into drug research. In 2014, the European Union introduced a gender toolkit for EU‐funded research, providing guidance on integrating gender into study design and analysis, though enforcement remains a challenge.^28^

In 2024, a Swiss government report underscored the urgent need to integrate sex‐ and gender‐sensitive considerations comprehensively into healthcare policy, research and education. The report identified key challenges, including insufficient research on female‐specific diseases, gender bias in diagnostics and treatment, and gaps in preventive measures tailored to women's health. Proposed measures include strengthening gender‐health expertise, embedding gender considerations into existing healthcare initiatives, and systematically educating healthcare professionals on gender‐sensitive healthcare needs.^29^

Further reinforcing this commitment, Switzerland's revised clinical trials law, effective November 2024, will mandate greater gender balance in research. Swissmedic, the national regulatory agency, has been tasked with enhancing the evaluation of sex and gender differences in new medicines by 2029.^30^ However, the role of research ethics committees (RECs) in addressing gender biases remains a critical issue, particularly in ensuring equitable representation of women, including pregnant women, in clinical research. Accordingly, there have been increasing calls for improved gender training, balanced gender representation within RECs, and clear guidelines to ensure that ethical reviews systematically incorporate sex and gender considerations.^31^

In 2020, swissethics—the umbrella organization of the Cantonal Ethics Committees in Switzerland, responsible for overseeing the ethical review and approval of clinical research involving human participants—issued recommendations for conducting sex‐ and gender‐equitable research. A dedicated working group further refined these guidelines, outlining how to integrate sex and gender considerations ethically into research. As a result, applicants are now required to address sex and gender equity in their ethics submissions.^32^

In medical education, recent studies emphasize that integrating sex‐ and gender‐sensitive medicine requires addressing not only explicit curricular content but also the implicit, or ‘hidden,’ curriculum, which often perpetuates stereotypes and biases. This includes diversity‐sensitive simulation training, inclusive language and imagery, and structural and institutional practices that shape how students learn about sex and gender.^33^ In 2022, eight medical faculties in Germany launched a collaborative initiative to systematically embed gender‐sensitive medicine into their curricula. Their approach includes revising educational content, training faculty, and integrating gender considerations into research and clinical practice. This initiative highlights the need for coordinated action among institutions to effectively address gender biases and achieve more equitable healthcare outcomes in Germany.^34^

Similarly, the University of Zurich established its first professorship dedicated exclusively to gender medicine, with another chair currently in development at the University of Bern.^35^ The Swiss Gender Health Network, a national organization committed to advancing sex‐ and gender‐sensitive medicine in Switzerland, has successfully integrated gender medicine into Swiss medical curricula. As of September 2024, it became an officially recognized society, furthering its mission to promote gender‐sensitive healthcare education and research.^36^ A major milestone was also achieved in 2023 with the launch of the National Research Program (NFP 83) by the Swiss Federal Council, marking the government's first large‐scale financial investment into research specifically focused on sex and gender differences in healthcare.^37^ Similar initiatives have emerged in Germany, Austria, Italy and internationally, including Israel, Japan and the United States, with many of these efforts supported and connected through the International Society for Gender Medicine.^38^

In recent years, several leading journals have enhanced transparency in how sex and gender considerations are integrated into study design and analysis. For example, researchers submitting manuscripts to Nature journals are now required to specify whether sex and gender analyses were conducted, provide disaggregated data by sex and gender where relevant, and justify their approach if such analyses were omitted.^39^ These measures aim to improve research accuracy and accountability, addressing historical oversights that have contributed to disparities in drug safety and efficacy between sexes. In addition, several academic journals have established Diversity, Equity, and Inclusion (DEI) editorial roles aimed at promoting inclusive research practices. These positions can help ensure that sex and gender considerations are systematically addressed during peer review and editorial evaluation, thereby fostering more equitable and representative biomedical research.

Clinically, major advancements have been observed globally, particularly in cardiology, where specialized ‘Women's Heart Centers’ have been established to address persistent inequities in the diagnosis and treatment of cardiovascular diseases in women.^40^ These centers have gained considerable recognition in the United States, both publicly and within the medical community. Europe, while somewhat behind in this regard, is making steady progress. Switzerland, for instance, not only hosts a Women's Heart Center^41^ but is also advancing gender‐sensitive approaches in other medical fields. In Zurich, psychiatrists have introduced an innovative therapeutic model specifically designed for men experiencing depression, developed through rigorous research.^42^ Most recently, a gender‐sensitive outpatient clinic in internal medicine was established in Magdeburg, Germany, further underscoring the growing momentum for gender‐adapted care across disciplines.^43^

**FIGURE 2:**
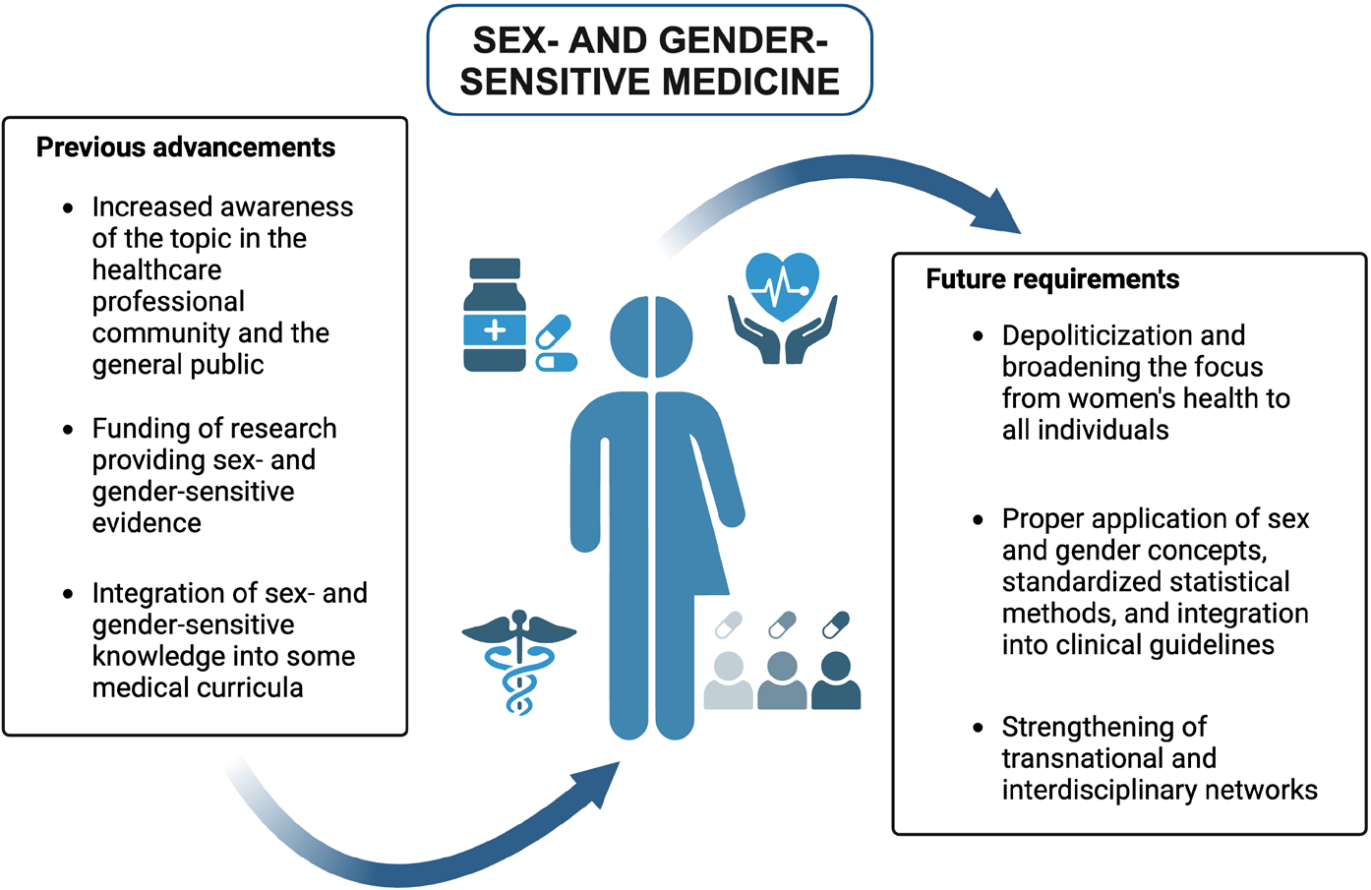
Key advancements in gender medicine and the necessary steps for future progress.

Despite these significant advancements and increasing international collaboration, substantial challenges remain. The systematic integration of sex and gender considerations into everyday clinical practice across medical disciplines is still incomplete, raising crucial questions about the barriers that continue to hinder widespread adoption. Figure 2 presents a concise overview of the key advancements in gender medicine and the necessary steps for future progress.

## 4 FROM BIAS TO BEST PRACTICE: ADDRESSING SEX AND GENDER IN RESEARCH

One of the primary barriers to integrating gender medicine into clinical practice is the reliance of Western medicine on evidence‐based guidelines, which are predominantly derived from large‐scale studies and meta‐analyses. Although there has been an increase in observational studies and small interventional trials addressing sex and gender differences, large‐scale studies in this field remain limited.^44^, ^45^ In many cases, subgroup analyses fail to investigate sex and gender differences, relegate them to supplementary materials or apply incorrect statistical methodologies.^46^, ^47^ A major conceptual challenge in the field is the frequent interchangeable use of the terms ‘sex’ and ‘gender.’ Many studies report either sex or gender, but not both, and often without clear definitions.^48^, ^49^ This practice contributes to inconsistent reporting, impairs reproducibility and obscures the biological and sociocultural factors that influence health outcomes. A rigorous distinction between sex (biological attributes) and gender (sociocultural roles and identities) is essential to ensure methodological clarity and interpretability.

The misapplication of subgroup analyses, leading to misleading interpretations, is a common challenge in medical statistics, but investigating sex and gender differences presents unique methodological hurdles.^50^ For instance, instead of treating sex as a covariate in multivariable regression models—an approach that increases susceptibility to confounding—differences between male and female study participants should be examined using a stratified approach.^51^ The goal of sex and gender analysis is to promote rigorous, reproducible, and responsible science. Additionally, there is growing recognition that the misconception that analysing sex‐specific effects significantly increases sample sizes and costs in biomedical research must be addressed. For example, a recent analysis demonstrated the potential benefits of a factorial study design using male and female animal models.^52^ The study found that including both sexes in experimental setups without explicitly testing for sex effects required minimal additional resources, emphasizing that sex‐specific analyses can be conducted without excessive effort. Pooling data across sexes assumes no difference between males and females, thereby preventing researchers from assessing the influence of sex on experimental outcomes.^53^ An increasing body of literature demonstrates that systematically integrating sex and gender analysis into scientific and engineering research enhances discovery, improves reproducibility and promotes social equity.^53^ Incorporating sex and gender considerations into experimental design has driven advancements across multiple disciplines, including improved heart disease treatment and better understanding of algorithmic bias in artificial intelligence.^53^ Conversely, neglecting sex and gender differences in biomedical research presents a significant public health risk. Notable examples include Alzheimer's treatment and heart disease, where sex and gender substantially influence disease risk, diagnosis, and treatment outcomes.^54^ Ignoring these factors not only leads to ineffective treatments and increased healthcare disparities but may also reinforce biases in artificial intelligence models increasingly used in medical research and practice.^55^

A compelling example of the consequences of disregarding sex and gender differences can be seen in the case of atrial fibrillation (AF). Women with AF face a significantly higher stroke risk than men, particularly after the age of 75.^56^, ^57^, ^58^ While the underlying reasons remain unclear, it is speculated that women on oral anticoagulation therapy spend more time outside the therapeutic range than men, potentially diminishing its protective effect.^59^

Despite well‐documented evidence of increased stroke risk in women with AF, the 2024 ESC Guidelines for the management of atrial fibrillation, developed in collaboration with the European Association for Cardio‐Thoracic Surgery (EACTS), removed female sex from the CHA_2_DS_2_‐VASc risk score, the most widely used stroke risk stratification tool in Europe. The guideline committee justified this decision by arguing that female sex is an age‐dependent stroke risk modifier rather than an independent risk factor.^60^ Additionally, concerns were raised that incorporating sex complicates clinical practice for healthcare professionals and patients, while also excluding individuals who identify as non‐binary, transgender, or those undergoing sex hormone therapy. However, this decision has sparked debate, as it raises concerns that prioritising inclusivity considerations may come at the cost of recognising specific stroke risks in biological women. Notably, a study published alongside the guidelines in support of this change excluded patients with a prior stroke or those aged ≥75, further complicating the interpretation of its conclusions.^48^

This example highlights the ongoing debate in healthcare regarding the importance assigned to sex and gender considerations. Some argue that these factors hold limited clinical relevance, yet understanding the pathophysiological mechanisms underlying sex‐based differences is crucial for recognizing the complexities that drive distinct disease manifestations in male and female patients. As demonstrated by atrial fibrillation, adopting a broader, sex‐ and gender‐inclusive perspective would not only deepen our understanding of disease processes but also improve outcomes for millions of patients worldwide. Acknowledging these challenges, there is growing momentum among funders and scientific journals to mandate the inclusion of sex as a biological variable in research, underscoring its significance for scientific rigor, reproducibility and clinical relevance.^61^

## 5 ADVANCING GENDER MEASUREMENT IN RESEARCH: THE DEVELOPMENT OF GENDER SCORES

Investigating gender as a variable presents unique statistical challenges. One major issue is the widespread misunderstanding of the distinction between ‘sex’ and ‘gender,’ leading to frequent misinterpretations in research.^62^ Even when gender is correctly conceptualized, defining it as a variable remains difficult due to the absence of international standards that ensure consistent interpretation and comparability of results.^63^

To address this, we and others have developed methodologies to identify key gender‐related variables in Western societies. One widely used approach is the creation of ‘gender scores,’ which condense multiple gender‐related factors into a single numerical variable for use in risk prediction models.^64^, ^65^, ^66^, ^67^, ^68^, ^69^ Similar to biological sex as a variable,^64^, ^67^ gender scores offer a methodological advantage by simplifying statistical modelling. Instead of incorporating multiple individual variables, which can reduce statistical power and complicate result interpretation, a single gender score allows for streamlined analysis.

One of the earliest gender scores, developed by Canadian researchers, integrated over 50 gender‐related variables into a single numerical value ranging from 0 (masculine) to 100 (feminine). This score was applied to the GENESIS‐PRAXY cohort, a study examining clinical endpoints in over 1000 patients with acute coronary syndrome.^70^ Their findings demonstrated a strong association between gender, cardiovascular risk factors, and clinical outcomes. The gender score incorporates variables that historically differ between men and women,^71^, ^72^ including education, parental and marital status, income, household responsibilities, perceived stress levels, childcare responsibilities and self‐assessed masculinity or femininity using the BEM Sex‐Role Inventory Scale.

Despite their utility, existing tools for assessing gender have limitations. For instance, in a cohort of 3000 SARS‐CoV‐2‐positive individuals in Switzerland, single components of a gender score were significant predictors of Post‐COVID‐19 Syndrome, whereas the overall gender score itself was not.^69^ This highlights the need for optimized instruments tailored for clinical and population research, particularly for use in large‐scale health surveys that reflect the growing diversity of European populations.

There is a pressing need in medical research and statistics to clearly distinguish between sex and gender, develop standardized tools for their consistent application, and commit to conducting stratified analyses from the outset. Standardized and validated gender scores will play a crucial role in improving the accuracy and applicability of gender‐related findings in healthcare research.

## 6 GENDER MEDICINE IN THE 21ST CENTURY: MORE THAN WOMEN'S HEALTH

A significant misconception hindering the broader acceptance and recognition of gender medicine is the tendency to equate it solely with women's health. This narrow perspective overlooks the crucial impact of sex and gender differences on men in various medical fields, including psychiatry, rheumatology and endocrinology.^73^ Such misconceptions can lead to reduced engagement with the field, particularly among male practitioners. Additionally, popular media often frames the gender health gap primarily through a feminist lens, reinforcing the perception that sex‐ and gender‐based medicine is a political rather than a scientific issue.^74^ While this framing played a role in the early development of gender medicine alongside the women's health movement, the field today extends far beyond women's health concerns.

Another critical yet often overlooked aspect of the gender health gap includes the unique health challenges faced by transgender and gender‐diverse individuals. Although they represent a smaller proportion of the population, their healthcare needs are distinct and require specific attention.^75^, ^76^ However, due to the high variability within this population, conducting rigorous studies remains difficult, often resulting in treatment guidelines that rely more on expert opinion than on robust empirical evidence. Addressing these disparities and ensuring equitable healthcare for all gender identities remains a significant challenge for the future of gender medicine.

## 7 ADVANCING GENDER MEDICINE: A ROADMAP FOR PROGRESS

The continued integration of gender medicine requires a coordinated, interdisciplinary approach across research, education, clinical practice and policy. While significant progress has been made, sustained collaboration is essential to ensure gender medicine becomes a standard component of healthcare rather than a niche specialty. Ignoring sex and gender differences leads to oversimplified health assessments, increased morbidity, and rising healthcare costs.

Depoliticizing gender medicine and broadening its scope beyond women's health is crucial. While feminist perspectives have historically shaped the field, the focus must shift toward evidence‐based, inclusive healthcare for all individuals. Future guidelines must embrace modern societal realities and ensure equitable health outcomes.

However, recent political developments in the United States, including the rollback of DEI programs in several states, threaten to undermine progress toward gender equity in healthcare and biomedical research. These policy shifts risk limiting funding opportunities, restricting inclusive education efforts, and marginalizing historically underrepresented groups. In this context, it is more important than ever to uphold the scientific imperative for independent, methodologically sound investigation of sex and gender differences in health and disease. Continued international collaboration and institutional commitment are critical to safeguarding evidence‐based progress in gender medicine.

**FIGURE 3:**
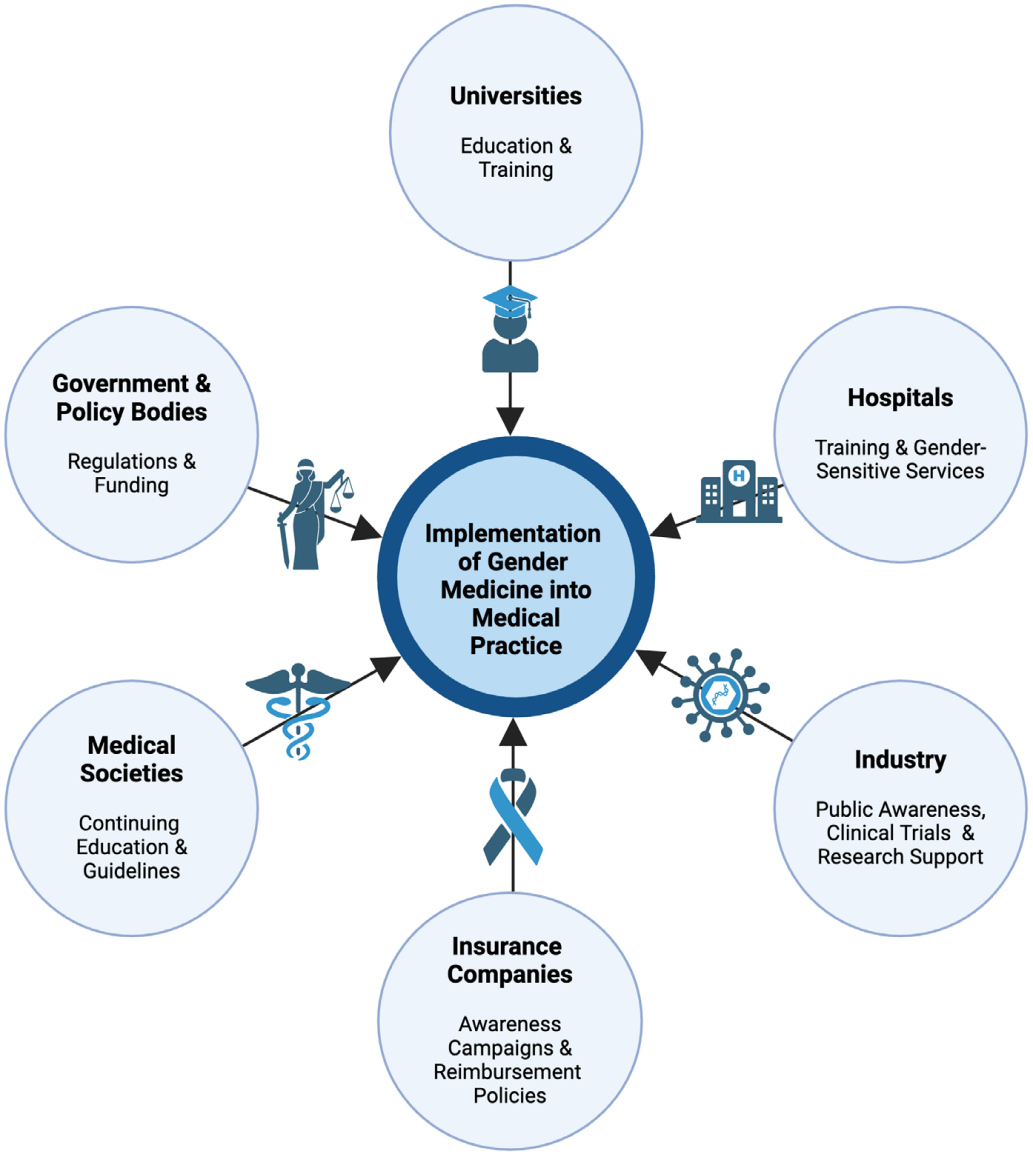
Interdisciplinary efforts required to implement gender medicine into medical practice, emphasizing the role of various actors in the healthcare system.

Against this backdrop, a structured action plan is essential to ensure long‐term implementation and resilience (Figure 3). Governments and regulatory bodies should fund gender‐sensitive research and enforce quality assessments that incorporate gender considerations. Universities must strengthen gender medicine education through dedicated professorships and specialised training. Hospitals need to integrate gender‐sensitive services and train healthcare professionals accordingly. Medical societies must develop guidelines that incorporate sex and gender perspectives, while insurers should assess the coverage and cost‐effectiveness of gender‐sensitive medical services. Industry should support public awareness campaigns and advance gender‐sensitive medical technologies.

Additionally, addressing gender biases in clinical practice is critical. A recently published ‘Toolkit for Reflection on Gender Bias in Medical Practice’ provides a framework to help healthcare professionals recognize and mitigate gender biases in areas such as patient interactions, diagnosis and treatment.^77^ By fostering awareness and encouraging self‐reflection, the toolkit promotes equitable, evidence‐based healthcare that accounts for biological and sociocultural determinants of health.

Ultimately, sex and gender differences do not need to be actively sought—they are inherently present. The key is acknowledging them and integrating this knowledge into healthcare. Standardizing research methodologies and strengthening global networks will pave the way for more effective, gender‐sensitive clinical guidelines. As Tom Robbins aptly stated, ‘Equality is not in regarding different things similarly; equality is in regarding different things differently.’

## FUNDING INFORMATION

CG was supported by grants from the Swiss National Science Foundation (SNSF) and the University of Zurich (UZH) Foundation. CEG was supported by an unrestricted research grant from the Intensive Care Unit (ICU) Research Foundation of the University Hospital Basel.

## CONFLICT OF INTEREST STATEMENT

There are no conflicts of interest related to the study design or its results.

## Data Availability

Anonymised data will become available to interested parties for non‐commercial reasons after the publication upon reasonable requests made to the corresponding author. Data requestors will need to sign a data access agreement.

## References

[1] Patwardhan V , Gil GF , Arrieta A , et al. Differences across the lifespan between females and males in the top 20 causes of disease burden globally: a systematic analysis of the global burden of disease study 2021. Lancet Public Health. 2024;9(5):e282‐e294. 38702093 10.1016/S2468-2667(24)00053-7 PMC11080072

[2] Regitz‐Zagrosek V , Gebhard C . Gender medicine: effects of sex and gender on cardiovascular disease manifestation and outcomes. Nat Rev Cardiol. 2023;20:236‐247. 36316574 10.1038/s41569-022-00797-4 PMC9628527

[3] McCarthy CR . Historical background of clinical trials involving women and minorities. Acad Med. 1994;69(9):695‐698. 8074757 10.1097/00001888-199409000-00002

[4] White J , Tannenbaum C , Klinge I , Schiebinger L , Clayton J . The integration of sex and gender considerations into biomedical research: lessons from international funding agencies. J Clin Endocrinol Metab. 2021;106(10):3034‐3048. 34137862 10.1210/clinem/dgab434 PMC8475217

[5] Nobelius AM , Wainer J . Gender and Medicine: A Conceptual Guide for Medical Educators. Monash University School of Rural Health; 2004.

[6] Legato MJ . Cardiovascular disease in women: what's different? What's new? What's unresolved? Ann N Y Acad Sci. 1994;736:147‐157. 7710201 10.1111/j.1749-6632.1994.tb12827.x

[7] Healy B . The Yentl syndrome. N Engl J Med. 1991;325:274‐276. 2057027 10.1056/NEJM199107253250408

[8] Killien M , Bigby JA , Champion V , et al. Involving minority and underrepresented women in clinical trials: the National Centers of excellence in Women's health. J Womens Health Gend Based Med. 2000;9(10):1061‐1070. 11153102 10.1089/152460900445974

[9] Daemmrich A . A tale of two experts: thalidomide and political engagement in the United States and West Germany. Soc Hist Med. 2002;15(1):137‐158. 12625358 10.1093/shm/15.1.137

[10] Legato MJ . Eve's rib: the groundbreaking guide to Women's health. Open Road Media; 2014.

[11] McGregor AJ , Templeton K , Kleinman MR , Jenkins MR . Advancing sex and gender competency in medicine: sex & gender women's health collaborative. Biol Sex Differ. 2013;4(1):11. 23724943 10.1186/2042-6410-4-11 PMC3674893

[12] Walther A , Schneeberger M , Eggenberger L . Evaluation of male‐specific psychoeducation for major depressive disorder compared to cognitive behavioral therapy psychoeducation: a randomized controlled investigation in mentally distressed men. Psychother Res. 2024;1‐18. Online ahead of print. 10.1080/10503307.2024.2398085 39257054

[13] Rice C , Harrison E , Friedman M . Doing justice to intersectionality in research. Cultural Studies ↔ Critic Methodol. 2019;19(6):409‐420.

[14] Heidari S , Babor TF , De Castro P , Tort S , Curno M . Sex and gender equity in research: rationale for the SAGER guidelines and recommended use. Res Integr Peer Rev. 2016;1:2. 29451543 10.1186/s41073-016-0007-6 PMC5793986

[15] Van Epps H , Astudillo O , Del Pozo Martin Y , Marsh J . The sex and gender equity in research (SAGER) guidelines: implementation and checklist development. Eur Sci ed. 2022;48:e86910. 10.12771/emj.2024.e11 PMC12093626 40703401

[16] Cox CE . Go Red for Women: Nearly 20 Years of Much Progress, Some Setbacks: Cardiovascular Research Foundation. 2023 Available from: https://www.tctmd.com/news/go‐red‐women‐nearly‐20‐years‐much‐progress‐some‐setbacks

[17] Usselman CW , Lindsey ML , Robinson AT , et al. Guidelines on the use of sex and gender in cardiovascular research. Am J Physiol Heart Circ Physiol. 2024;326(1):H238‐H255. 37999647 10.1152/ajpheart.00535.2023 PMC11219057

[18] Soldin OP , Mattison DR . Sex differences in pharmacokinetics and pharmacodynamics. Clin Pharmacokinet. 2009;48(3):143‐157. 19385708 10.2165/00003088-200948030-00001 PMC3644551

[19] Mauvais‐Jarvis F , Berthold HK , Campesi I , et al. Sex‐ and gender‐based pharmacological response to drugs. Pharmacol Rev. 2021;73(2):730‐762. 33653873 10.1124/pharmrev.120.000206 PMC7938661

[20] Wilson LAB , Zajitschek SRK , Lagisz M , Mason J , Haselimashhadi H , Nakagawa S . Sex differences in allometry for phenotypic traits in mice indicate that females are not scaled males. Nat Commun. 2022;13(1):7502. 36509767 10.1038/s41467-022-35266-6 PMC9744842

[21] Zucker I , Prendergast BJ . Sex differences in pharmacokinetics predict adverse drug reactions in women. Biol Sex Differ. 2020;11(1):32. 32503637 10.1186/s13293-020-00308-5 PMC7275616

[22] Watson S , Caster O , Rochon PA , den Ruijter H . Reported adverse drug reactions in women and men: aggregated evidence from globally collected individual case reports during half a century. EClinicalMedicine. 2019;17:100188. 31891132 10.1016/j.eclinm.2019.10.001 PMC6933269

[23] Buckley RF , Gong J , Woodward M . A call to action to address sex differences in Alzheimer disease clinical trials. JAMA Neurol. 2023;80(8):769‐770. 37155156 10.1001/jamaneurol.2023.1059 PMC11446568

[24] Lindholt JS , Søgaard R , Rasmussen LM , et al. Five‐year outcomes of the Danish cardiovascular screening (DANCAVAS) trial. N Engl J Med. 2022;387(15):1385‐1394. 36027560 10.1056/NEJMoa2208681

[25] Gaudino M , Fremes SE , Mehran R , Bairey Merz CN . ROMA:women: innovative approaches for the first cardiac surgery trial in women. Circulation. 2023;148(17):1289‐1291. 37009730 10.1161/CIRCULATIONAHA.123.064033

[26] Femtech is transforming women's healthcare. But it must include everyone Davos, Switzerland: World Economic Forum. 2023 Available from: https://www.weforum.org/stories/2023/05/femtech‐healthcare‐bipoc/

[27] World Economic Forum . Closing the Women's Health Gap: A $1 Trillion Opportunity to Improve Lives and Economies. 2024 Available from: https://www.weforum.org/publications/closing‐the‐women‐s‐health‐gap‐a‐1‐trillion‐opportunity‐to‐improve‐lives‐and‐economies/

[28] Commission E, Research D‐Gf, Innovation . Gender in EU‐funded research – Toolkit. Publications Office; 2014.

[29] Amacker M , Büchler T , Bigler C , Nydegger K . Schlussbericht: Gesundheit der Frauen. Bessere Berücksichtigung ihrer Eigenheiten; 2024:86.

[30] Swissmedic . Better representation of women in medical research Switzerland. 2024 Available from https://www.swissmedic.ch/swissmedic/en/home/news/mitteilungen/beruecksichtigung‐frauen‐med‐forschung.html

[31] Saxena A , Lasher E , Somerville C , Heidari S . Considerations of sex and gender dimensions by research ethics committees: a scoping review. Int Health. 2022;14(6):554‐561. 35043198 10.1093/inthealth/ihab093 PMC9623496

[32] Swissethics . publishes recommendations on research adapted to “sex and gender”. Swiss Association of Research Ethics Committees; 2024.

[33] Wortmann L , Oertelt‐Prigione S . Teaching sex‐ and gender‐sensitive medicine is not just a matter of content. J Med Educat Curri Develop. 2024;11:23821205241304531. 10.1177/23821205241304531 PMC11622302 39650070

[34] Haserueck A . Geschlechtersensible Medizin: Acht medizinische Fakultäten kooperieren. Dtsch Arztebl; 2023:1.

[35] Gendermedizin . Medizinische Fakultät | UZH. University of Zurich; 2024.

[36] Gender and Medicine: A Network of Swiss Universities. Unisanté; 2024.

[37] Schibler B , Schoenholzer S , Leuthold J . National Research Programme (NRP) 83: Gender Medicine and Health Bern: Swiss National Science Foundation. 2025. Available from: https://www.nfp83.ch/en

[38] The International Society for Gender Medicine (IGM) . Available from: https://www.intgsm.org/.

[39] Raising the bar on sex and gender reporting in research. Nat Commun. 2022;13(1):2845. 35585146 10.1038/s41467-022-30398-1 PMC9117190

[40] Khandelwal A , Bakir M , Bezaire M , et al. Managing ischemic heart disease in women: role of a Women's heart center. Curr Atheroscler Rep. 2021;23(10):56. 34345945 10.1007/s11883-021-00956-x PMC8331213

[41] Gebhard C . Frauenherzzentrum. Department of Cardiology, Inselspital University Hospital Bern; 2023. Available from: https://kardiologie.insel.ch/de/unser‐angebot/frauenherzzentrum.

[42] Eggenberger L , Ehlert U , Walther A . New directions in male‐tailored psychotherapy for depression. Front Psychol. 2023;14:1146078. 37143589 10.3389/fpsyg.2023.1146078 PMC10151934

[43] Seeland U . Geschlechtersensible Medizin mit Hochschulambulanz Magdeburg: University of Magdeburg. 2025. Available from https://gsm.med.ovgu.de/

[44] Jacobs EG . Only 0.5% of neuroscience studies look at women's health. Here's how to change that. Nature. 2023;623:667. 37989773 10.1038/d41586-023-03614-1

[45] Meynet P , Improta R , Carbone ML , et al. Percutaneous coronary intervention versus coronary artery bypass grafting in left main disease according to patients' sex: a meta‐analysis. Eur J Clin Investig. 2025;55(2):e14348. 39543458 10.1111/eci.14348 PMC11744918

[46] Aulakh AK , Anand SS . Sex and gender subgroup analyses of randomized trials. Womens Health Issues. 2007;17(6):342‐350. 17936640 10.1016/j.whi.2007.04.002

[47] Gebhard C , Regitz‐Zagrosek V . Colchicine in patients with chronic coronary disease. N Engl J Med. 2021;384(8):776‐777. 10.1056/NEJMc2034992 33626261

[48] Champsi A , Mobley AR , Subramanian A , et al. Gender and contemporary risk of adverse events in atrial fibrillation. Eur Heart J. 2024;45(36):3707‐3717. 39217497 10.1093/eurheartj/ehae539 PMC11439109

[49] Teppo K , Airaksinen KEJ , Jaakkola J , et al. Temporal trends of gender disparities in oral anticoagulant use in patients with atrial fibrillation. Eur J Clin Investig. 2024;54(1):e14107. 37823410 10.1111/eci.14107

[50] Sun X , Briel M , Walter SD , Guyatt GH . Is a subgroup effect believable? Updating criteria to evaluate the credibility of subgroup analyses. BMJ. 2010;340:c117. 20354011 10.1136/bmj.c117

[51] Schenck‐Gustafsson K , D PR , Pfaff DW , Pisetsky DS . Handbook of Clinical Gender Medicine. S.Karger AG; 2012.

[52] Buch T , Moos K , Ferreira FM , Fröhlich H , Gebhard C , Tresch A . Benefits of a factorial design focusing on inclusion of female and male animals in one experiment. J Mol Med (Berl). 2019;97(6):871‐877. 30980104 10.1007/s00109-019-01774-0

[53] Tannenbaum C , Ellis RP , Eyssel F , Zou J , Schiebinger L . Sex and gender analysis improves science and engineering. Nature. 2019;575(7781):137‐146. 31695204 10.1038/s41586-019-1657-6

[54] Haupt S , Carcel C , Norton R . Neglecting sex and gender in research is a public‐health risk. Nature. 2024;629(8012):527‐530. 38750229 10.1038/d41586-024-01372-2

[55] Zack T , Lehman E , Suzgun M , et al. Assessing the potential of GPT‐4 to perpetuate racial and gender biases in health care: a model evaluation study. Lancet Digit Health. 2024;6(1):e12‐e22. 38123252 10.1016/S2589-7500(23)00225-X

[56] Zeitler EP , Poole JE , Albert CM , et al. Arrhythmias in female patients: incidence, presentation and management. Circ Res. 2022;130(4):474‐495. 35175839 10.1161/CIRCRESAHA.121.319893

[57] Wagstaff AJ , Overvad TF , Lip GY , Lane DA . Is female sex a risk factor for stroke and thromboembolism in patients with atrial fibrillation? A systematic review and meta‐analysis. QJM. 2014;107(12):955‐967. 24633256 10.1093/qjmed/hcu054

[58] Fang MC , Singer DE , Chang Y , et al. Gender differences in the risk of ischemic stroke and peripheral embolism in atrial fibrillation: the AnTicoagulation and risk factors in atrial fibrillation (ATRIA) study. Circulation. 2005;112(12):1687‐1691. 16157766 10.1161/CIRCULATIONAHA.105.553438 PMC3522521

[59] Pokorney SD , Simon DN , Thomas L , et al. Patients' time in therapeutic range on warfarin among US patients with atrial fibrillation: results from ORBIT‐AF registry. Am Heart J. 2015;170(1):141‐148. 26093875 10.1016/j.ahj.2015.03.017

[60] Van Gelder IC , Rienstra M , Bunting KV , et al. 2024 ESC guidelines for the management of atrial fibrillation developed in collaboration with the European Association for Cardio‐Thoracic Surgery (EACTS). Eur Heart J. 2024;45(36):3314‐3414. 39210723 10.1093/eurheartj/ehae176

[61] Willingham E . The fraught quest to account for sex in biology research. Nature. 2022;609(7927):456‐459. 36100679 10.1038/d41586-022-02919-x

[62] Rioux C , Paré A , London‐Nadeau K , et al. Sex and gender terminology: a glossary for gender‐inclusive epidemiology. J Epidemiol Community Health. 2022;76:764‐768. 10.1136/jech-2022-219171 35725304

[63] Nielsen MW , Stefanick ML , Peragine D , et al. Gender‐related variables for health research. Biol Sex Differ. 2021;12(1):23. 33618769 10.1186/s13293-021-00366-3 PMC7898259

[64] Pelletier R , Ditto B , Pilote L . A composite measure of gender and its association with risk factors in patients with premature acute coronary syndrome. Psychosom Med. 2015;77(5):517‐526. 25984818 10.1097/PSY.0000000000000186

[65] Lacasse A , Pagé MG , Choinière M , et al. Conducting gender‐based analysis of existing databases when self‐reported gender data are unavailable: the GENDER index in a working population. Can J Public Health. 2020;111(2):155‐168. 31933236 10.17269/s41997-019-00277-2 PMC7109207

[66] Smith PM , Koehoorn M . Measuring gender when you don't have a gender measure: constructing a gender index using survey data. Int J Equity Health. 2016;15(1):82. 27233478 10.1186/s12939-016-0370-4 PMC4884354

[67] Pelletier R , Khan NA , Cox J , et al. Sex versus gender‐related characteristics: which predicts outcome after acute coronary syndrome in the young? J Am Coll Cardiol. 2016;67(2):127‐135. 26791057 10.1016/j.jacc.2015.10.067

[68] Gebhard CE , Hamouda N , Gebert P , Regitz‐Zagrosek V , Gebhard C . Sex versus gender‐related characteristics: which predicts clinical outcomes of acute COVID‐19? Intensive Care Med. 2022;48(11):1652‐1655. 35943570 10.1007/s00134-022-06836-5 PMC9361238

[69] Gebhard CE , Sütsch C , Gebert P , et al. Impact of sex and gender on post‐COVID‐19 syndrome, Switzerland, 2020. Euro Surveill. 2024;29(2):2300200. 38214079 10.2807/1560-7917.ES.2024.29.2.2300200 PMC10785203

[70] Leung Yinko SS , Pelletier R , Behlouli H , Norris CM , Humphries KH , Pilote L . Health‐related quality of life in premature acute coronary syndrome: does patient sex or gender really matter? J Am Heart Assoc. 2014;3(4):e000901. 25074696 10.1161/JAHA.114.000901 PMC4310372

[71] Nauman AT , Behlouli H , Alexander N , et al. Gender score development in the Berlin aging study II: a retrospective approach. Biol Sex Differ. 2021;12(1):15. 33461607 10.1186/s13293-020-00351-2 PMC7814714

[72] Pohrt A , Kendel F , Demuth I , et al. Differentiating sex and gender among older men and women. Psychosom Med. 2022;84:339‐346. 35149636 10.1097/PSY.0000000000001056

[73] Hankivsky O . Women's health, men's health, and gender and health: implications of intersectionality. Soc Sci Med. 2012;74:1712‐1720. 22361090 10.1016/j.socscimed.2011.11.029

[74] Keane H . Feminism and the complexities of gender and health. Aust Fem Stud. 2014;29:180‐188.

[75] Pellicane MJ , Ciesla JA . Associations between minority stress, depression, and suicidal ideation and attempts in transgender and gender diverse (TGD) individuals: systematic review and meta‐analysis. Clin Psychol Rev. 2022;91:102113. 34973649 10.1016/j.cpr.2021.102113

[76] T'Sjoen G , Arcelus J , Gooren L , Klink DT , Tangpricha V . Endocrinology of transgender medicine. Endocr Rev. 2019;40(1):97‐117. 30307546 10.1210/er.2018-00011

[77] Geiser E , Clair C , Schwarz J . Checklist: A toolkit for reflection on gender bias in medical practice.

